# High performance *in silico* virtual drug screening on many-core processors

**DOI:** 10.1177/1094342014528252

**Published:** 2015-05

**Authors:** Simon McIntosh-Smith, James Price, Richard B Sessions, Amaurys A Ibarra

**Affiliations:** 1 Department of Computer Science, University of Bristol, Bristol, UK; 2 School of Biochemistry, University of Bristol, Bristol, UK

**Keywords:** Molecular docking, *in silico* virtual drug screening, many-core, GPU, OpenCL, performance portability

## Abstract

Drug screening is an important part of the drug development pipeline for the pharmaceutical industry. Traditional, lab-based methods are increasingly being augmented with computational methods, ranging from simple molecular similarity searches through more complex pharmacophore matching to more computationally intensive approaches, such as molecular docking. The latter simulates the binding of drug molecules to their targets, typically protein molecules. In this work, we describe BUDE, the Bristol University Docking Engine, which has been ported to the OpenCL industry standard parallel programming language in order to exploit the performance of modern many-core processors. Our highly optimized OpenCL implementation of BUDE sustains 1.43 TFLOP/s on a single Nvidia GTX 680 GPU, or 46% of peak performance. BUDE also exploits OpenCL to deliver effective performance portability across a broad spectrum of different computer architectures from different vendors, including GPUs from Nvidia and AMD, Intel’s Xeon Phi and multi-core CPUs with SIMD instruction sets.

## 1 Introduction

*In silico* molecular docking is a computational technique for predicting the structure of a complex formed between two molecules and estimating the strength of their interaction (Halperin et al., 2002). Until recently, the computational cost of applying this method to libraries of millions of candidate drug molecules (or *ligands*) has been prohibitive, as each ligand-protein docking is itself a computationally expensive operation. With the relentless march of Moore’s Law, however, this technique is becoming increasingly important to the pharmaceutical industry. Halperin et al. (2002) observed that docking is computationally challenging because of the many different ways in which two molecules may be arranged together to form a complex (three translational and three rotational degrees of freedom), while Shoichet et al. (1992) observed that the number of the potential arrangements between the two molecules being docked grows exponentially with the size of the components. Further, interacting all patches of the surface of one protein molecule with all patches of a second molecule requires on the order of 10^7^ trials, each one of which is a computationally expensive operation (Cherfils and Janin, 1993).

More traditional virtual screening approaches use simplified representations (pharmacophores) of the candidate ligands and sometimes part of the protein surface. This allows very rapid selection or filtering of extremely large datasets of candidate drug molecules. These kinds of approaches can also be used for detecting molecular similarity between known binders and candidate ligands, ranging from simple properties that can be coded into bit-strings (Tanimoto fingerprints (Willett, 2006)), through more detailed information coded into pharmacophores (Leach et al., 2010), to detailed descriptions of the electric field around molecules (Cheeseright et al., 2008). As computer performance and methodologies advance, we can envisage molecular docking augmenting or even replacing traditional virtual screening methods. We are currently near a crossing point where the number of available drug-like molecules (1 – 2 × 10^7^ compounds) can now be screened by docking in just a few days using HPC systems. Growth in known drug-like chemical space is expected to be slower than growth in computer performance, hence attention can be focused back onto improving the accuracy of predictions. In turn, rapid (traditional) virtual screening methods will be able to probe the vast, untapped chemical space of unknown but tractable small molecules yet to be synthesized (Reymond et al., 2010). Likewise, more accurate calculation of binding free energies (Woods et al., 2011) can be applied to the small number of ligands (*O*(10^2^)) identified as most promising by molecular docking techniques.

### 1.1 The overall aims and achievements of our work

In this work, we present an optimized version of BUDE, the Bristol University Docking Engine. BUDE enables true *in silico* virtual drug screening by docking, by exploiting a unique combination of techniques:

- a genetic algorithm-based search of the six degrees of freedom in the arrangement of the protein and drug molecules, which evaluates only a small fraction of the overall search space;
- extremely fast implementations of the docking and scoring computational kernels using the OpenCL parallel programming language to exploit the latest CPU and GPU hardware;
- a tuned empirical free-energy forcefield for predicting the binding pose and energy of the ligand with the target protein.

BUDE was one of the first applications to be adapted for modern day accelerators. A port to ClearSpeed’s CSX parallel architecture was demonstrated on a cluster of 120 CSX600 accelerators on the exhibition floor at the International Conference for High Performance Computing, Networking, Storage and Analysis in Reno in 2007 (a cluster so energy efficient it was able to run off a small battery backup system for five minutes during a power cut). Up to 12 CSX600 accelerators could be packed into a 1U ClearSpeed Accelerated Terascale Server (CATS) chassis. A single 1U CATS system achieved a BUDE speedup of 21× over an at-the-time contemporary dual socket, dual core (four cores total) 2.6 GHz x86-based 1U server (McIntosh-Smith and Sessions, 2008). BUDE was later ported to GPUs in 2010, when it was used to compare the performance and energy efficiency of a range of different CPUs and GPUs, with an Nvidia C2050 delivering a speedup of 4.0× compared to an eight core (dual socket, quad core) x86 system (McIntosh-Smith et al., 2012).

The new contributions presented in this paper are:

1. Highly optimized computational kernels for the docking and scoring functions using OpenCL, which are capable of sustaining a significant fraction of the hardware’s peak performance. We believe the performance we have achieved is amongst the highest sustained performance for any real application on a GPU.
2. Techniques exploiting OpenCL to enable *performance portability* across a wide range of different computer architectures, including CPUs, GPUs and accelerators. The results we present demonstrate that OpenCL can enable effective performance portability across a diverse range of parallel architectures, an increasingly important requirement in HPC, especially during the current proliferation of CPU, GPU and accelerator architectures.
3. An in-depth analysis of the performance of our molecular docking application, BUDE, comparing performance across a diverse range of the latest performance-oriented processors from Intel, Nvidia and AMD.

### 1.2 Related work

Due to the value of molecular docking in terms of discovering or designing new potential drugs, a wide range of different molecular docking codes have been developed (Muegge and Rarey, 2003; Fan et al., 2009). As yet, only a relatively small subset of molecular docking applications have been ported to use many-core high performance architectures, such as GPUs or Intel’s Xeon Phi.

Van Court et al. (2004) undertook one of the earliest projects to accelerate a molecular docking program, when they ported ZDOCK to FPGAs in 2004. They achieved a speedup of about 200× for the 3D FFT portion of the code, when compared to a single CPU core (Van Court et al., 2004). Subsequent projects also explored porting docking codes to FPGAs (Sukhwani and Herbordt, 2008). After these early explorations with FPGAs, and not long after the first port of BUDE to a many-core architecture (ClearSpeed’s CSX architecture in 2006), other projects explored porting docking codes to the emerging many-core architectures, such as IBM’s Cell processor (May, 2008; Servat et al., 2008). Sukhwani and Herbordt (2009) at Boston University were, in 2009, among the first to adopt GPUs for molecular docking, porting the PIPER production code and achieving a 6.1× speedup compared to optimized code on a quad core CPU. PIPER was followed by a number of GPU ports for other docking codes (Kannan and Ganji, 2010; Macindoe et al., 2010; Korb et al., 2011; Simonsen et al., 2011).

Our own work accelerating BUDE on many-core architectures differs from that described above in a number of key areas. First, we have ported BUDE’s entire functionality to the accelerator, only leaving the initial application startup and final shutdown on the host. Most of the previous work ported just the top few computationally intensive functions to the accelerator. Second, BUDE’s port has been designed to be performance portable across a wide variety of many-core architectures, including GPUs from multiple vendors, Intel’s Xeon Phi, and even multi-core CPUs with wide SIMD instruction sets. This is in contrast to previous projects which have tended to focus on FPGAs or GPUs from one vendor. Third, BUDE has received extensive optimization, and can sustain over 40% of floating point peak performance on a wide range of different architectures. To the best of our knowledge, this high level of optimization exceeds that of all other molecular docking codes, and indeed, BUDE is sustaining performance at a level only exceeded by very few other codes of *any* kind (vendor-optimized BLAS being one of the few such examples). This combination of a full port of all functionality to the accelerator, performance portability and a very high level of optimization, sets BUDE apart from previous work in porting molecular docking codes to many-core architectures.

Performance portability is becoming an increasingly important research area in HPC, as the range of processor architectures continues to diversify, and the effort required to port an application to run efficiently on a many-core platform can be significant. In the early days of GPU programming, the scientific software community had no choice but to use proprietary languages such as Nvidia’s CUDA (Nvidia, n.d.). With the emergence and maturing of open standards for many-core programming, such as OpenCL (Khronos Group, n.d.), developers now have more flexible options open to them which avoid proprietary lock-in to one vendor. Today’s HPC applications ideally need to be able to run efficiently on multi-core CPUs with SIMD instruction sets, many-core GPUs, heterogeneous APUs, and even more exotic hardware such as accelerators and FPGAs. OpenCL is one of the few parallel programming models available today that has been developed with both performance portability and heterogeneous computing as design goals. A 2013 study by Zhang et al. (2013) investigated how OpenCL programs can be parametrized in order to optimize performance on different target hardware, including CPUs with SIMD instruction sets, CPUs with integrated GPUs, and discrete GPUs. In 2011, Fang et al. (2011) performed an in-depth study across 16 benchmarks of the performance penalty of using a platform portable parallel programming language such as OpenCL, over a platform specific equivalent, such as CUDA. They concluded that, while the platform specific language had some advantages in certain circumstances, on the whole OpenCL’s platform portability had little to no detrimental impact on its performance. In 2012, Du et al. (2012) and Agullo et al. (2009) presented their findings for a performance portable version of the triangular solver and matrix multiply kernels from the MAGMA BLAS/LAPACK library. They exploited OpenCL in combination with autotuning techniques to deliver performance portability across a range of GPUs from Nvidia and AMD. In 2012, Kim et al. (2012) described their performance portable platform for OpenCL programs called SnuCL. This framework exploits OpenCL to deliver performance portability across heterogeneous hardware, and across multiple devices. At around the same time, Pennycook et al. (2013) examined the performance portability of an OpenCL implementation of LU from the NAS Parallel Bench Suite (Bailey et al., 1991) and saw that, with appropriate autotuning techniques, it was possible for the OpenCL implementation to achieve performance competitive with native FORTRAN 77 and CUDA implementations running on the same hardware, while Herdman et al. (2012) compared the performance of a Lagrangian-Eulerian explicit hydrodynamics mini-application and found that OpenCL delivered similar performance to CUDA and OpenACC. Finally, the PEPPHER European FP7 project (The PEPPHER Consortium, n.d.), at the time of writing, is exploring the performance portability of parallel and heterogeneous programs, reporting a comparison of three different approaches, all of which could make use of OpenCL to deliver the required portability (Kessler et al., 2012).

## 2 Background

### 2.1 BUDE: Docking procedure

**Figure 1.**
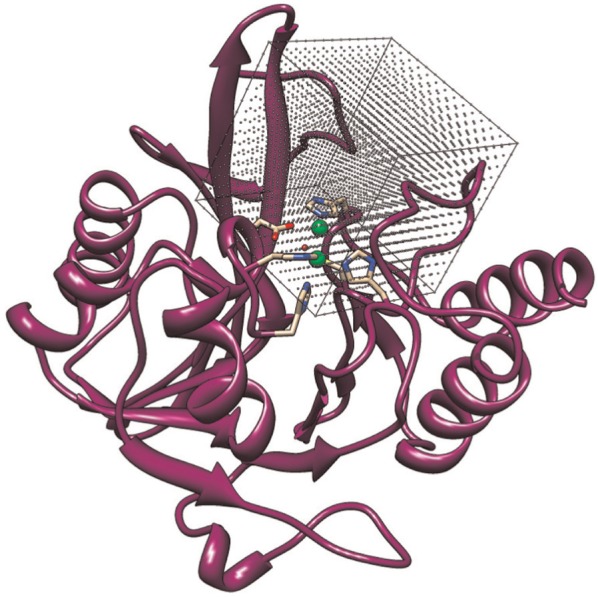
**The EMC search space around the active site of an NDM-1 protein molecule.** Each point in the grid represents a potential pose, which has a position and rotation in three dimensional space.

**Figure 2.**
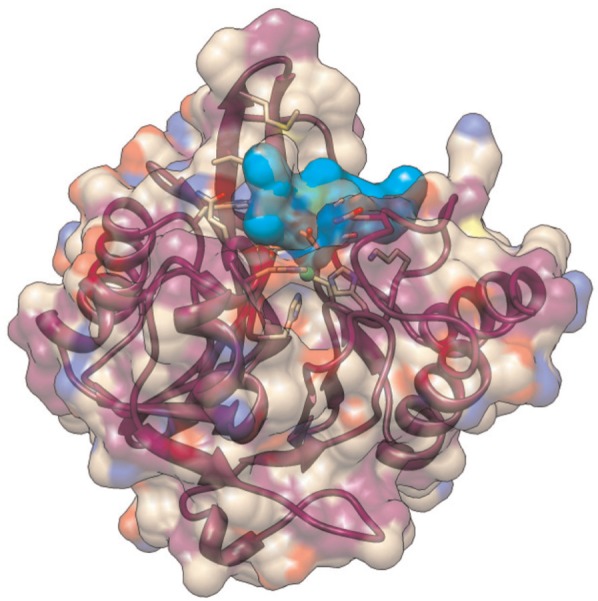
**The predicted structure of a ligand docked to an NDM-1 protein molecule.** The ligand is shown in turquoise, with the protein in purple.

**Figure 3.**
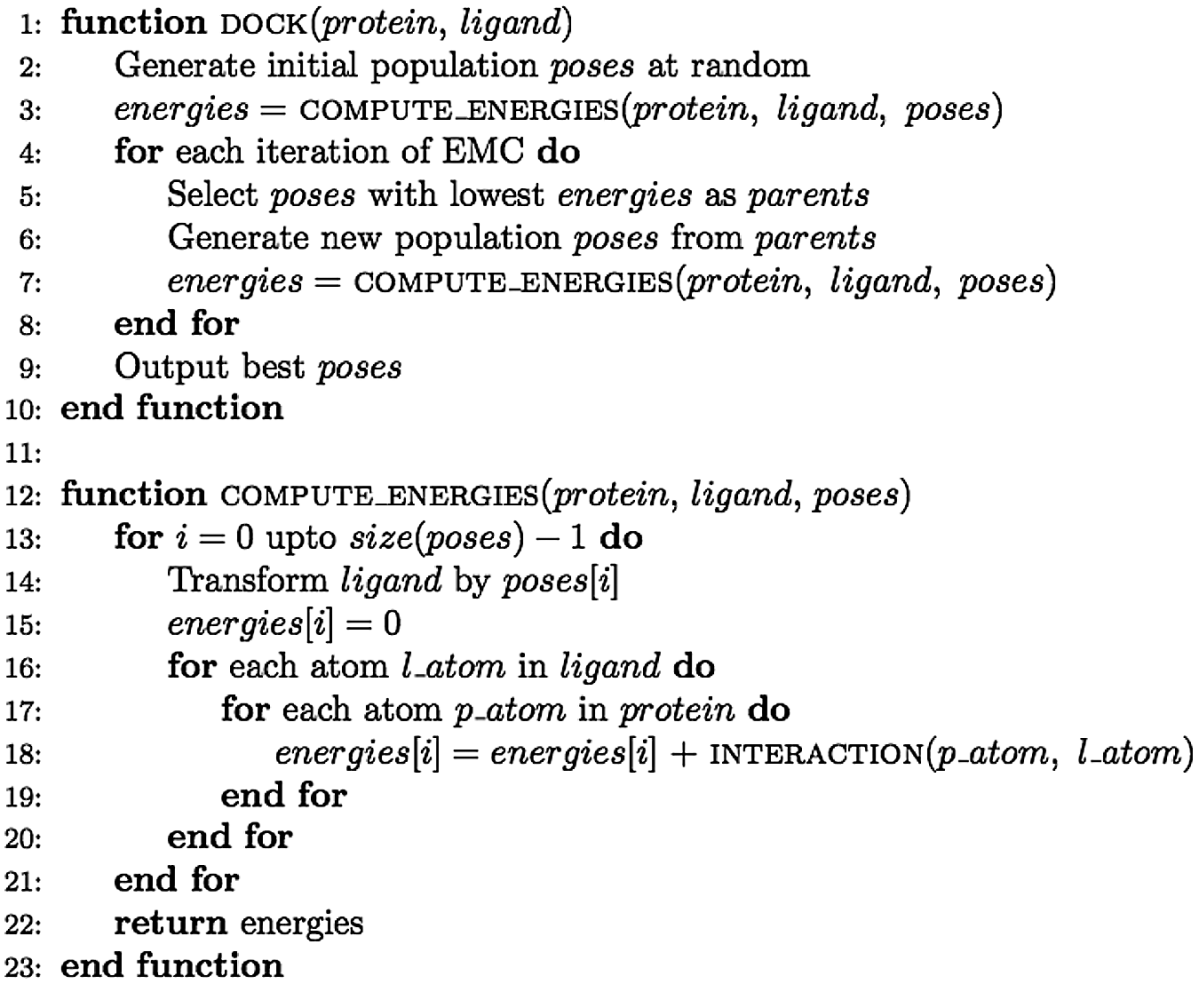
Pseudo-code description of the docking algorithm employed by BUDE.

The energy minimization algorithm employed by BUDE is based on the Evolutionary Monte Carlo (EMC) techniques described by Gibbs et al. (2001). The problem domain is defined as a six dimensional search space across the potential positions and rotations that the ligand can take relative to the target molecule, also known as the receptor. A single point in this domain is referred to as a “pose”, which is indexed by the pose’s *transformation descriptor*. The search comprises a sequence of generations, each of which involves evaluating the binding energy between the receptor and the ligand in a number of poses (the population). Figure 1 shows the search space around the active site of an NDM-1 protein molecule. The first generation evaluates a population of poses generated uniformly at random over the search space, and each subsequent generation uses a population containing poses which are randomly mutated from some number of the best poses in the previous generation. The algorithm terminates after a fixed number of generations; the number of iterations is determined experimentally and is chosen so that it is large enough for the population to converge. Figure 2 shows a ligand docked to the active site of an NDM-1 protein molecule with a high binding affinity. A pseudo-code representation of BUDE’s algorithm is given in Figure 3.

For a detailed explanation of the set of equations that make-up BUDE’s empirical free energy forcefield, the interested reader is referred to McIntosh-Smith et al. (2012). BUDE’s atom-based forcefield is derived from the coarse-grained forcefield developed for protein folding with RAFT (Gibbs et al., 2001). The molecular interaction energy calculated by BUDE approximates to a free energy of binding.

BUDE’s ‘soft-core’ forcefield is designed to accommodate the geometric approximation inherent in the method of rigid-body docking of a relatively small number of ligand variations, or *conformations* (1–50 per compound), on a relatively coarse grid (typically 1Å grid spacing and 10° rotations). The functions are simple to calculate but computationally challenging since they are discontinuous and need to be evaluated many times.

The critical characteristic of the equations that constitute BUDE’s forcefield is that they naturally employ a large degree of *conditional behavior*. Indeed, of the 50 or so operations per atom–atom interaction, 20% of these would be branches (the remaining operations are single precision floating point operations such as adds, multiplies, reciprocals and square roots). This is a very high ratio of branches for an application we wish to optimize for a many-core architecture. What is more, many of these branches are conditionally based on the distances between the two atoms under test, and so the branches are likely to be highly divergent; that is, when the code is executed in a data-parallel manner, there is a high probability of conditional branches in different ‘lanes’ of the data parallelism wanting to execute down different paths, causing both paths of the branch to be executed. In general we wish to avoid branching in data parallel code as the control flow hazards they generate severely impact the performance of many-core pipelines. Divergent branches are even worse, as not only do they disrupt the efficient use of the pipeline, but they cause both paths of an ‘if-else’ to be executed.

### 2.2 The OpenCL programming model

When deciding which parallel programming language to use for BUDE, we had a number of design goals we wished to meet. First, we wanted a language which would work across all the hardware platforms we might wish to target. Rewriting BUDE was a major undertaking that has taken several years to bring to fruition, so we did not want to resort to using a language that would only work on a small subset of our target hardware. Second, we wanted a language which would allow us to express all the natural parallelism in the BUDE docking application. BUDE is solving a naturally very parallel problem, and so we wanted to be able to express this parallelism in the new implementation. We therefore required support for different levels of parallelism, including both data and task level parallelism. Finally, we wanted a parallel language that made it as easy as possible for us to incrementally port our existing code, rather than having to rewrite everything from scratch. Based on these design goals, we analyzed all of the available parallel programming languages, including OpenCL, CUDA, OpenACC, Threaded Building Blocks and CILK. Only one clearly met all of our criteria: OpenCL.

OpenCL is an open standard for cross-platform parallel programming (Khronos Group, n.d.). Its central premise is to provide the programmer with a means to expose the parallelism in their application in order to exploit the highly parallel nature of modern computer hardware. The portions of the program that will execute in parallel are contained in kernels which define the computation for a single element in the problem domain. A single instance of this kernel is known as a work-item. Work-items can be grouped together into work-groups, with work-items in the same work-group able to synchronize with one another and share a small amount of local memory. At runtime, the application can launch a one, two or three dimensional grid of work-items to execute the kernel across the full problem domain in parallel. The programming language used for OpenCL kernels is a modified version of C99.

OpenCL includes a conceptual model for the hardware on which it will run. The primary compute component in the OpenCL hardware model is an OpenCL device, for example a GPU or CPU. Devices consist of one or more compute units (CUs), each of which contains one or more processing elements (PEs). The PEs within one CU operate in a data parallel fashion. Work-groups execute on a single CU, while work-items are executed on PEs. Work-items within a work-group can share access to the CU’s local memory. All work-items can access a device’s global memory. For more details on OpenCL’s programming model, see Khronos Group (n.d.), Munshi et al. (2011) and Gaster et al. (2011).

OpenCL is widely supported by most parallel hardware vendors. There are optimized implementations available for CPUs from Intel and AMD, optimized implementations for GPUs from Nvidia, AMD, ARM, Imagination Technologies, Intel and Qualcomm, and an implementation for Intel’s Xeon Phi, amongst others.

### 2.3 Nvidia’s Kepler architecture

The primary GPU targeted in this work was an Nvidia GTX 680 (Nvidia, 2012a), based on the GK104 variant of the Kepler architecture. This GPU contains 1536 single precision floating point units, which Nvidia terms CUDA cores. These are grouped together into 8 units of 192, called Streaming Multiprocessors, or SMXs. Programs are run on the Kepler architecture in a single instruction multiple threads (SIMT) style. These threads are executed in groups of 32, known as a warp. Threads within a warp execute instructions in lockstep. Each SMX unit contains four warp schedulers, and each of these schedulers can dispatch up to 2 independent instructions per cycle from a single warp. Each SMX can execute up to 6 different 32 element wide SIMD instructions at a time. When a warp stalls due to a high latency instruction (for example, a read from DRAM), the SMX will swap in another warp to maintain core utilization. Each SMX also contains a small amount of on-chip memory that can be shared between its CUDA cores. The GTX 680 is capable of 3.09 TFLOP/s theoretical peak performance (single precision).

Transcribing the GTX 680’s characteristics into OpenCL terminology, the SMX cores are CUs, the CUDA cores are PEs, and the SMX memory is local memory. A GTX 680 therefore has 1536 PEs grouped together into 8 CUs of 192 PEs each.

## 3 Methods

### 3.1 Overview

In this work we had two specific goals. First, we wanted to discover just what fraction of peak performance it was possible to sustain for a real molecular docking application, BUDE, on a specific target GPU, the Nvidia GTX 680. Second, we wanted to explore performance portability for our highly optimized BUDE implementation, and in particular to discover whether OpenCL would enable us to have a single implementation which would perform well on a wide variety of highly parallel computer architectures. This performance portability investigation was particularly interesting to us, because many of the GPU optimization studies performed to date have focused on only a specific platform, such as CUDA on an Nvidia Tesla GPU, or OpenMP with MPI on an Intel Xeon Phi. The ability to port an application once and then have it run fast everywhere is extremely attractive to software developers, and the maturing of the OpenCL standard represents a unique opportunity to develop a cross-platform many-core application.

### 3.2 Optimization approach

The evaluation of free energy for a population of poses (the compute_energies function in Figure 3) represents the vast majority of the computation required to dock a ligand molecule with a receptor molecule, and this functionality was implemented as a single, highly optimized OpenCL kernel. A typical BUDE *in silico* virtual drug screening run will require docking every ligand from a database of millions of molecules, each consisting of around 15–40 non-hydrogen atoms. Receptor molecules are typically much larger, consisting of a few thousand atoms. To dock a single ligand conformation to a receptor we need to evaluate the binding energies for hundreds of thousands of poses. Therefore to process an entire database of millions of virtual ligands, we will need to calculate the docking energies of potentially billions of poses. Each pose can be evaluated independently of the others, and we exploit this data parallelism in OpenCL by assigning each pose to a separate work-item. Compared to the previously published version of BUDE (McIntosh-Smith et al., 2012), in this new work we focused on significantly improving the throughput of the primary kernel and in porting the remaining computation from the host to the OpenCL device. We have achieved significant performance improvements, first by considering the memory access patterns the primary kernel exhibits, and then by heavily optimizing the instructions used for energy calculations to maximize the utilization of the GPU’s floating point units.

The individual optimizations that we have made in this work are not unique in themselves. However, the extent to which we have optimized BUDE’s code, successfully eliminating *all* of the branches from what is otherwise a naturally highly branch-dependent code, is one novel aspect of this work. Our approach involved analyzing the assembly code being generated for BUDE’s computational kernels on a specific architecture, before going back to modify the higher level OpenCL code in order to assist the compiler in generating the best possible output. This is an extreme form of code optimization which, to the best of our knowledge, has not been applied to a molecular docking code before. In addition, we have avoided the potential pitfall of a code which is highly optimized for just a small range of target devices. Instead we have achieved a resulting code which is both highly optimized *and* highly performance portable across a diverse range of many-core architectures. This is in contrast to most prior work in this area, which tended to focus on optimizations for just one specific architecture or range of GPUs from one vendor.

### 3.3 Memory access patterns

Our previous implementation of BUDE (McIntosh-Smith et al., 2012) assigned forcefield parameters to atoms during initialization, packing these parameters together with the position of each atom into a single structure. The motivation for this original design was that this assignment of values into a per-atom structure would be performed only once, after which the parameters would be readily available in each atom’s data structure during energy evaluation. However, the resulting atom structure was 40 bytes in size, suboptimal for most memory subsystems, which are typically optimized for memory accesses which are multiples of powers of two in size. By instead performing parameter assignment on demand inside the energy evaluation kernel, we were able to reduce the atom structure to a position (three 32-bit floats) and type (one 32-bit integer). This new atom structure was just 16 bytes in size, which aligned much more efficiently with most hardware’s memory interfaces.

The forcefield used in this work comprised seven different parameters, and was defined for approximately 35 atom types. With each parameter stored as a single-precision floating point number or a 32-bit integer, the resulting look-up table was ~1 KByte in size. This allowed us to explicitly copy the forcefield into the GPU’s fast on-chip (local) memory, instead of relying on caches to minimize the latency of retrieving forcefield parameters from DRAM. This optimization mitigated the cost of repeatedly assigning forcefield parameters within the energy evaluation kernel and yielded small performance gains over pre-assigning parameters into packed atom structures. We also noticed that the forcefields we used typically had some parameters that were equal for all 35 atom types (e.g. interaction cut-off distances). For these cases, we were able to exploit a form of metaprogramming, removing the parameters from the look-up table, and instead building them into the kernel as constant values that were set when the OpenCL kernel was compiled at runtime.

Each work-item operated on a different pose, and therefore requested a separate six-float transformation descriptor (TD) before computing the resulting transformation matrix used to calculate each ligand atom’s position and orientation. Our previous implementation stored these TDs as contiguous 24 byte blocks in an array-of-structures (AoS) data layout. In this new work we optimized the code to instead use a structure-of-arrays (SoA) format, which in turn enabled more efficient, coalesced memory accesses in very parallel memory subsystems, such as those found in GPUs and in the Xeon Phi. On the GTX 680 which was our focus for this work, overlapping the memory accesses to these data structures with arithmetic instructions allowed the SMX warp-scheduler to hide the DRAM access latency with computation, and using SoA format for the TDs increased the coalescence of the memory accesses, resulting in a higher overall throughput and a more efficient use of the memory subsystem.

### 3.4 Instruction sequence optimization

As described at the end of Section 2.1, the equations which constitute BUDE’s forcefield contain many branches, and thus the evaluation of atom–atom energies naturally exhibits a high degree of conditional behavior: each forcefield function only contributes an energy value to the binding affinity for certain combinations of atom type and pairwise distance. Since groups of 32 work-items (warps) execute in a SIMD fashion on Kepler, these work-items are sensitive to divergent branches, during which some PEs may sit idle while other PEs in the same warp are executing a divergent branch body. Even conditional branches that are uniform, that is, where all the work-items in the same warp branch the same way, will have a large and typically detrimental impact on performance, as GPUs tend to have very simple branch predictors and deep pipelines. In these circumstances, even in the best case a branch will cause pipeline stalls and lose performance. To alleviate the performance impact of conditional branches in general, and divergent branches in particular, the inner loop body of the energy evaluation kernel was rewritten to transform conditional branches into combinations of predicated selection and multiplication to achieve semantically equivalent behavior, but without the control flow.

**Figure 4.**
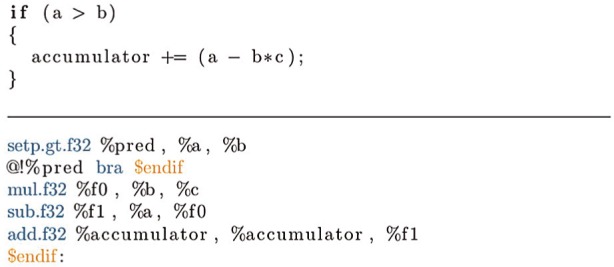
**Conditional accumulation of an expression.** Shown in OpenCL C, with the corresponding assembly code (Nvidia PTX).

**Figure 5.**
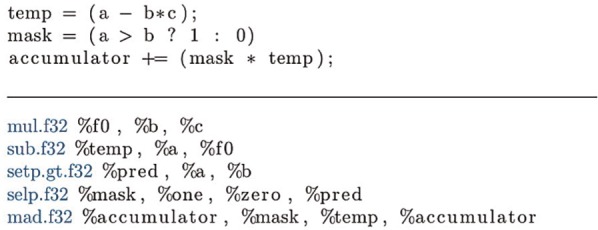
**Predicated accumulation of an expression.** Shown in OpenCL C, with the corresponding assembly code (Nvidia PTX).

For example, the conditional accumulation shown in Figure 4 can be replaced with the code in Figure 5 which multiplies the expression by zero if the condition is not met. While both examples compile to the same number of instructions, the latter removes the overhead of branching by unconditionally evaluating the expression. Another benefit of the predicated approach is that it exposes some instruction level parallelism (the *sub* and *setp* instructions can be executed independently), which can be exploited by the dual-issue capability of the Kepler architecture. Replacing the branches with predicated code had the additional benefit of increasing the size of the basic block with which the compiler could schedule instructions, enabling it to make more efficient use of the instruction pipeline, with fewer pipeline stalls caused by a lack of appropriate instructions that can be issued and executed in parallel.

Parallel thread execution (PTX) is an intermediate assembly language used by Nvidia as a proprietary, device-agnostic assembly code representation for GPU kernels (Nvidia, 2012b). In order to identify which lines of code were causing the OpenCL kernel compiler to generate branch instructions, we generated the PTX output for our primary OpenCL kernel by passing the CL_PROGRAM_BINARIES flag to the clGetProgramInfo() OpenCL command. By analyzing the PTX and identifying which parts of the kernel were causing branches to be generated, we were able to incrementally modify the code, replacing each branch-generating sequence in the kernel with a semantically equivalent sequence which was more amenable to predicated execution. Proceeding in this manner, we were able to successfully remove all branch instructions from the inner loop of the kernel, significantly improving GPU utilization and overall performance. Guided by our analysis of the PTX, we also made further changes to enable the compiler to generate single-cycle multiply add (FMA/MAD) instructions where possible.

**Figure 6.**
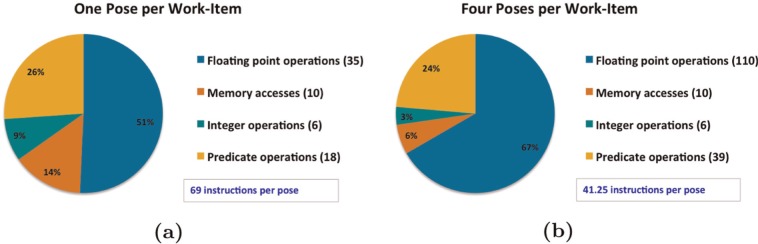
**Instruction mix for the innermost loop in the energy evaluation kernel.** (a) shows the mix for one pose per work-item, while (b) shows the impact of unrolling the pose loop four times.

**Figure 7.**
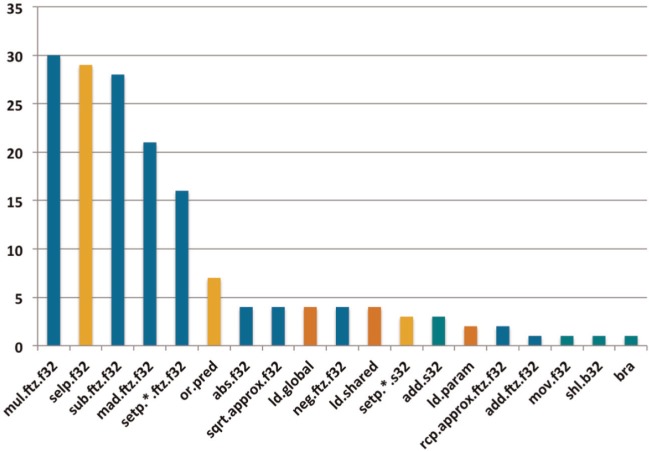
**PTX instruction histogram for innermost loop in energy evaluation kernel.** The *y*-axis indicates the number of instructions executed within the main energy evaluation kernel. Results shown are for the four poses per work-item scheme.

To increase data reuse within the OpenCL kernel, and thus increase arithmetic intensity and overall performance, the code was modified to process multiple poses within each work-item. This optimization meant that more energy evaluations could be performed for each protein atom loaded in the inner loop, which served to amortize the cost of loading atom data from DRAM. For the GTX 680, we found that processing four poses per work-item gave the highest performance. Figure 6 shows the instruction mix for the innermost loop of the kernel when computing a single pose per work-item compared to four poses per work-item. This approach reduced the number of memory accesses per pose by more than half. Instructions for pointer arithmetic and predicate generation were also amortized by this approach, leading to further performance gains. The vast majority of instructions in the final version of the kernel were floating point operations, and with the exception of the unavoidable branch at the end of the loop, all of the conditional behavior in the loop body had been transformed into predicated execution via use of the *setp* and *selp* PTX instructions; see Figure 7.

### 3.5 Host performance

Although the energy evaluation kernel was the performance critical component of BUDE, we had to keep Amdahl’s Law in mind, and ensure that, as we optimized this kernel, we were not merely moving the bottleneck to the generation and evolution of poses during the EMC. These additional parts of the docking process (lines 2, 5 and 6 in Figure 3) were therefore also ported to OpenCL in order to minimize their contribution to the overall runtime. This optimization removed the need for any data transfer between the host and OpenCL device other than the initial data (16 bytes per atom, 15–40 atoms per ligand, ~1000 atoms per receptor) and final results (six float transformation descriptors and a four byte ligand identifier per best result, ~100 best ligands returned), between them a trivial amount of data.

The generation and evolution of the population of poses used in the EMC relied on a random number generator (RNG). In order to efficiently generate random numbers in parallel, we produced an OpenCL implementation of *WarpStandard* (Thomas, n.d.), a GPU specific RNG that provides one generator per work-item (thread). This RNG was selected due to its balance of speed and statistical quality, and we verified that the accuracy of the docked structures predicted by BUDE was no worse when using this RNG.

To evolve a population of poses in the EMC, one needs to select the subset of poses from the current population that have the lowest energies (line 5 in Figure 3). The genetic algorithm in BUDE needs to select the best *K* elements from an unsorted list of size *N*, where *K* is *O*(10^2^) and *N* is *O*(10^5^). In the previous implementation of BUDE, this partial sorting operation was performed on the host using a variant of quicksort. The recursive nature of the quicksort algorithm means that it is not well suited for GPUs, and so a new approach was required in this work. Our solution to this sorting problem was based on an approach devised by Felipe Cruz at the University of Nagasaki, using a two stage algorithm.

In the first stage, we split the list into equal partitions of size *B* (which we call bins), where we chose *B* = 1024 to suit the target architecture. We assigned a work-group to each of the ⌈N/B⌉ bins, and used an implementation of Batcher’s bitonic merge sorting algorithm (Batcher, 1968; Knuth, 1998) to sort the contents of each bin, using local memory to store the intermediate results. We chose merge-sort for several reasons. The first reason is merge-sort’s low parallel time complexity of *O*(log^2^ *N*). Secondly, the merge-sort procedure is non-recursive so it is straight-forward to implement efficiently on a wide range of architectures, including our target GPU. Fast merge-sort implementations consist of a set of comparators with no data-access conflicts, an important performance consideration for very parallel memory subsystems, such as those found in GPUs and Xeon Phi (otherwise, conflicts in accessing local memory would occur, which would then be serialized).

In the second stage, after the merge-sort of the individual bins, we merge the sorted bins, but only needed the first *K* elements of the sorted list. To achieve this without needing to wastefully merge the bins completely, we ran a second kernel comprising a single work-group, with a work-item assigned to each bin. Each work-item examined a single element in their bin, and the work-item with the lowest element wrote this pose as output, before that work-item moved on to the next element. This step iterated *K* times, and we again used local memory for all of the intermediate data.

With all of the computation ported to the OpenCL device (in this case the GTX 680 GPU), we needed to address BUDE’s disk access to avoid this becoming the new bottleneck. At the beginning of a BUDE docking run, the data describing the target protein (receptor) and the database of ligands to be docked all begin on disk. Care was taken to minimize the data transfer requirements on the filesystem. A preprocessing step was introduced to parse the protein and ligand molecules from the text-based Tripos Mol2 format (Tripos, 2005) into the raw binary data required by the docking kernel. This reduced the average amount of data required to store a single ligand conformation from 1720 bytes to just 430 bytes, a reduction of 75%. This drastically reduced the strain on the filesystem during large screening runs, when all 160 million conformations of the 8 million ligands in the ZINC database would need to be processed. In our new binary format this still required ~69 GBytes of disk space. This final optimization also removed the need for the host to parse the atom data during screening, thus further reducing the load on the host.

## 4 Performance results

All of the GPU benchmarks presented in the following section were performed on the same host system, listed in Table 1. Our benchmark docked a total of 128 conformations from 10 different ligands in the ZINC database (UCSF, n.d.) to a target protein molecule (NDM-1, PDB code 3Q6X) with 938 atoms in the docking site. BUDE’s EMC docking process was set to consider eight generations, each requiring 65,536 poses to be evaluated. The primary metric for measuring performance was the number of atom–atom pairwise interactions computed per second (line 18 in Figure 3). This metric was independent of the size of the EMC and molecules, and bears some relation to other molecular modeling applications (although the amount of work to perform a single interaction will likely be very different for other docking codes).

**Table 1.** Benchmark system specifications.

	Nvidia	AMD	Intel
OpenCL device(s)	GTX 680, GTX 780 Ti, Tesla K20c	Radeon HD7970, Radeon R9 290X, FirePro S10000	Xeon Phi SE10P, Xeon E5-2687W (x2)
CPU model	Intel Core i5-3550	Intel Core i5-3550	Intel Xeon E5-2687W (x2)
CPU specs	4 cores, 3.3 GHz	4 cores, 3.3 GHz	16 cores, 3.1 GHz
Main memory	16GB	16GB	32GB
Operating system	Ubuntu 12.04	Ubuntu 12.04	RHEL Server 6.3
Driver/SDK	Driver 331.20	fglrx 13.25.5	Intel SDK for OpenCL Applications XE 2013 R3 (3.2.1.16712), MPSS 2.1.02.0390, Intel Fortran compiler (ifort) 13.1.0 20130121

In order to determine how well our code was utilizing the GPU, we also measured the number of FLOP/s performed. Since all conditional execution was removed from the inner loop of the optimized version of the energy evaluation kernel, the number of FLOPs executed when docking a particular ligand was dependent only on the number of atoms in the ligand and protein, and the number of poses in the EMC. Our baseline (original) version of the code contained data-dependent conditional execution however, and so we instrumented both versions of the docking kernel with a counter to calculate the total number of FLOPs that were actually performed. We counted each floating point arithmetic instruction as a single FLOP, including comparisons and special instructions (sqrt, sin and cos). The only exceptions were the FMA and MAD instructions, which we counted as two FLOPs each.

**Figure 8.**
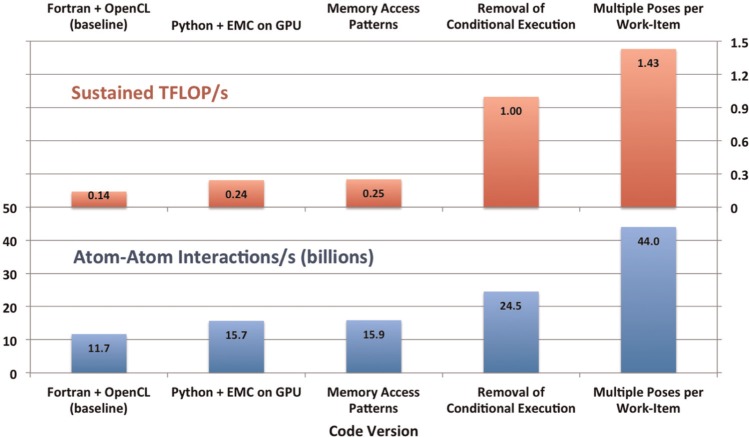
Effect of individual optimizations on performance and utilization on a GTX 680.

Figure 8 shows the incremental effects of our main optimizations to BUDE. Our baseline code for performance comparisons was our original OpenCL port of the energy evaluation kernel, with the remainder of the application running in Fortran (sequentially). This was the version of BUDE previously described in our work on measuring the energy efficiency of GPUs (McIntosh-Smith et al., 2012). All speedups are reported for the wall-clock time for the complete docking run, not simply for the kernel times. Also, all speedups are reported cumulatively, i.e. a speedup of 10% followed by another speedup of 10% represents a total speedup of 21%, not 20%, over the starting performance.

For this new work, we ported the small-ligand docking host code to Python, using PyOpenCL (Klöckner, n.d.) to interface with OpenCL. The EMC code previously running on the host was ported to the GPU during this conversion. During our experiments, we found that explicitly targeting an older version of the Nvidia architecture by adding -cl-nv-arch sm_13 to the OpenCL kernel build options yielded a 10% increase in performance. This is likely due to a difference in compiler technology: older versions of Nvidia’s toolchain used a proprietary compiler, but more recently they have migrated to LLVM. This newer toolchain will still be maturing, and therefore may not produce as optimal code as the previous compiler. Similar behavior has been observed by others (Kurzak et al., 2012). At this stage we also reordered the nesting of the protein and ligand loops in the energy kernel (lines 16 and 17 in Figure 3) in order to remove the need to precompute and store the transformed ligands. These improvements together yielded a total speedup of 34.2%.

Next we performed the modifications to memory layout, creating a forcefield look-up table and coalescing accesses to transformation data. At this stage in the optimization process, this optimization did not have as big an impact on performance as we initially expected, giving only a 1.7% increase in speed. After some investigation, we concluded that this was largely due to the majority of BUDE’s memory accesses being broadcasts from global memory: every work-item operated on the same atom data at the same time. This meant that the total cost of memory accesses was already small in the baseline code, and so optimizing this part of the code yielded a relatively small improvement to overall performance.

Replacing conditional branches with predicated execution and processing multiple poses per work-item had much more significant effects on both performance and device utilization. Replacing all the conditional branches with predicated execution yielded a 54.1% speedup, while the multiple poses per work-item optimization resulted in a further 79.6% performance improvement (both measured in atom–atom interactions per second). The combined benefit of these two optimizations resulted in a 2.8× speedup.

With all of these optimizations applied, we revisited the memory layout optimization in order to quantify its importance in supporting the later optimizations. Taking the final version of the code and then undoing just the memory layout optimization resulted in a 14.0% reduction in the sustained TFLOP/s and a 15.0% reduction in the delivered atom–atom interactions per second, proving that the memory layout optimization was critical to the eventual performance of the fully optimized code.

With all of these optimizations applied, our highly optimized code achieved 44 billion atom-atom interactions/s on the GTX 680, taking 37 s to dock the 128 conformations (~0.3 s per conformation). It achieved a sustained performance of 1.43 TFLOP/s when measured across the entire BUDE run, representing 46% of the peak single precision performance of the device. Compared to our baseline code, this improved device utilization by 10.2×, resulting in an overall increase to docking throughput of 3.8× (see Table 2). Just over 96% of the overall runtime was spent inside the energy evaluation kernel, and the amount of time the GPU was idle was negligible. It is interesting to observe how our modifications from conditional code to predicated code have affected performance and FLOP counts, the latter having to increase by ~10× to deliver a ~4× increase in the former. This seems a reasonable tradeoff; many-core processors have FLOPs in abundance, and we have successfully exploited them in this work to deliver significant real-world performance improvements.

**Table 2.** Relative speed-up for individual optimizations on a GTX 680.

Code version	Relative interactions/s	Relative TFLOP/s	Energy evaluation time (%)
Baseline	1.00	1.00	94
Python + EMC on GPU	1.34	1.74	99
Memory access patterns	1.36	1.79	99
Removal of conditional execution	2.09	7.11	98
Multiple poses per work-item	3.76	10.21	96

The 3.09 TFLOP/s of theoretical peak performance of the target GPU was computed on the basis that on every clock cycle, every processing element in the device can perform two single precision floating point operations (a multiply and an add). Clearly this peak performance is unlikely to be reached in reality; to achieve the theoretical peak performance an application would have to consist entirely of FMA/MAD instructions, with negligible overheads due to memory accesses and conditional behavior.

In order to understand our achievement of sustaining 46% of peak performance for BUDE, consider the GEMM set of matrix multiply subroutines in the Basic Linear Algebra Subprograms library (BLAS) (National Science Foundation and Department of Energy, n.d.). Matrix multiplication is a fundamental building block for many other scientific routines, and since it consists of a high degree of multiply and accumulate operations, it typically gets closer to peak performance than almost any other function. As such, GEMM is often used to benchmark hardware for HPC, and for example, DGEMM is the most performance critical subroutine in the LINPACK benchmark (Dongarra et al., n.d.) used to compile the TOP500 list of supercomputers (Meuer et al., n.d.). Recent performance results for single precision, complex matrix multiply (CGEMM) running on a GTX 680 show that this GPU can achieve up to 56% of peak performance, the highest sustained performance we have found in the literature for this GPU (Kurzak et al., 2012). The BUDE application is much more complex than matrix multiply: it contains complex conditional behavior, and some of the floating point operations in the inner loop are square roots and reciprocals. Since we were measuring performance across the whole application as opposed to a single kernel, we believe that our sustained performance of 46% is an excellent result and amongst the highest sustained performance achieved for a real application on a GPU.

### 4.1 Performance portability with OpenCL

**Figure 9.**
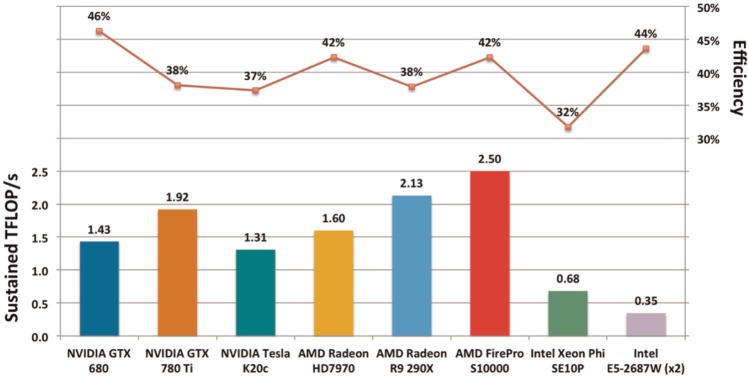
**Performance comparison across various devices.** Reported as sustained TFLOP/s on the bottom, and as a percentage of peak performance sustained on the top.

An important feature of the OpenCL framework is its portability; the same OpenCL code will run on devices from a wide variety of hardware vendors. One of the main goals of our work was to explore the performance portability potential of OpenCL. Figure 9 shows the maximum sustained FLOP/s that the new OpenCL version of BUDE presented in this paper achieved on a selection of devices, and the corresponding fraction of their theoretical peak floating point performance this represents. In the optimized version of BUDE, the docking throughput is roughly proportional to the sustained floating point performance; the sustained performance of 1.43 TFLOP/s on the GTX 680 translates to approximately 44 billion atom–atom interactions per second.

In all runs across all devices, all of the BUDE source code, including the OpenCL kernel code, was identical; the only change made when benchmarking each device was to tune three simple parameters. First, we chose the number of poses per work-item, typically four. Second, we chose the work-group size to suit each device. Third, we selected a total number of poses such that the total number of work-groups was exactly divisible by the number of compute units. Finally, we ensured that the total number of poses (and therefore the total problem size) was as close to 65,356 as possible. The systems used to benchmark each device are listed in Table 1.

Perhaps unsurprisingly, the highest fraction of peak performance was achieved on the GTX 680, as most of our optimizations were developed on this device. However, the same code also performed well across all the other devices in the test, averaging 40% efficiency (i.e. sustaining on average 40% of peak floating point performance across the whole application).

The standout device was the AMD S10000, which sustained 2.50 TFLOP/s for BUDE, 42% of its peak performance. This performance translates to an atom–atom interaction rate of 76.1 billion, 73% greater than the GTX 680. The other AMD GPUs also performed well for BUDE, with the Radeon R9 290X sustaining 2.13 TFLOP/s, a 38% efficiency, and the Radeon HD7970 sustaining 1.60 TFLOP/s at an efficiency of 42%. All of these GPUs outperformed our original target device, Nvidia’s GTX 680, even though they were executing the code as optimized for the latter device.

Nvidia’s recent high-end consumer GPU, the GTX 780 Ti, proved to be the fastest Nvidia device in the study, sustaining 1.92 TFLOP/s at an efficiency of 38%. The performance on the Nvidia K20c was perhaps a little lower than we expected. The K20c has a slightly higher peak single precision floating point performance than the GTX 680 (3.52 TFLOP/s vs 3.1 TFLOP/s), and yet it delivers a lower performance for BUDE, both in terms of absolute performance and as a percentage of peak (37% vs 46%). We believe part of the reason is that the K20 is only supported by the newer Nvidia drivers and SDK, and so is forced to use their newer, less mature LLVM-based compiler. We have already seen how this factor alone can result in a 10% performance decrease on the GTX 680. One can also observe that the degree of parallelism in a K20c is much larger than the GTX 680 (2496 PEs vs 1536), and so the K20 may require larger problem sizes to deliver greater performance.

The Intel Xeon Phi achieved a respectable efficiency of 32%, not far behind the Nvidia K20c’s 37%. There were a number of reasons for the Phi’s slightly lower performance than the other devices, including a lower single precision floating point peak performance, a younger and therefore less mature software stack, and the Xeon Phi having more significant architectural differences to the Nvidia GPU which had been our primary optimization target. It is possible that with further modifications to our kernel code we may have been able to improve its efficiency on Xeon Phi devices; we plan on pursuing this line of inquiry in future work. We have also observed significant improvements in the performance of the code being generated by Intel’s OpenCL implementations during the writing of this paper, and based on this experience, we would expect further improvements to come.

The Intel Xeon CPU impressively achieved a similar fraction of peak performance to the GTX 680 GPU when running the same OpenCL code (44%). The high percentage of peak performance that was sustained on the CPU indicated that Intel’s OpenCL SDK was able to successfully vectorize the main kernel, exploiting the CPU’s SIMD AVX instruction set. By comparison, the sequential Fortran version of BUDE was 2.6× slower than the OpenCL implementation in terms of atom–atom interactions per second when run on the same dual CPU configuration (with 32 concurrent instances employed by the Fortran version to utilize all of the hardware threads). The improved vectorization was likely due the OpenCL compiler having much more explicit information about the available parallelism than the Fortran compiler.

**Figure 10.**
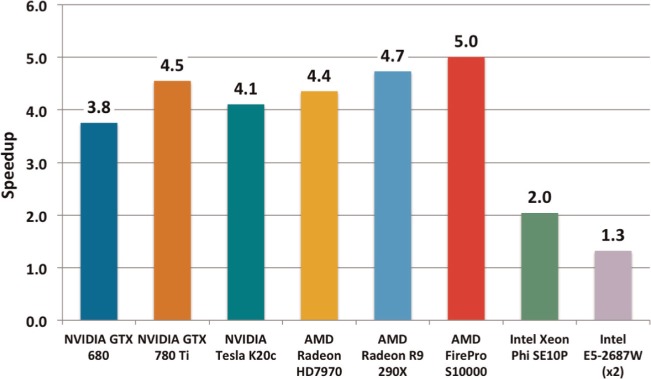
Performance improvements as a result of the optimizations developed in this work. Speedup is measured relative to the atom–atom interactions/s of the baseline BUDE code.

The optimizations for BUDE presented in this work improved its performance on the target device, the Nvidia GTX 680, by a factor of 3.8×. We thought it interesting to examine the effect of the optimizations on the other devices in this study. A chart of the impact of the optimizations, essentially showing the ‘before’ and ‘after’ cases, is shown in Figure 10.

Perhaps surprisingly, the optimizations developed for the GTX 680 actually had a *bigger* positive impact on most of the other devices in the study. The GTX 780 Ti saw the biggest improvement of the Nvidia GPUs, with a 4.5× increase over the baseline version of BUDE, while the AMD GPUs saw an average improvement of 4.7×, a significantly larger improvement than the GTX 680’s 3.8×. The Xeon Phi and Xeon CPU saw smaller gains from this work, seeing improvements of 2.0× and 1.3× respectively. Given the relatively high fraction of peak performance these devices are sustaining, this result suggests that the Xeon Phi and Xeon CPU were performing better, relative to the other devices, on the original version of BUDE.

As a final experiment, we re-implemented our OpenCL kernels as a direct port into CUDA and re-ran them on our Nvidia platforms. Interestingly, performance using CUDA was 5–10% worse than with OpenCL, despite the intermediate PTX code that was generated being very similar. Thus in this instance OpenCL has delivered the dual benefits of greater performance and performance portability.

### 4.2 Future work

We intend to investigate extending our use of metaprogramming to further improve the efficiency of the energy evaluation kernel. By pre-sorting the atoms of the receptor and/or ligand, we can group interactions that exhibit the same forcefield behavior together. Since these interactions will use the same functions for computing the energy, we can produce a simplified code sequence for them. We will then be able to generate a kernel at runtime that has specialized loop bodies for the most common atom types, only using the generalized form of the loop for the remaining atoms. This approach should result in an overall increase in docking throughput.

A further improvement we wish to test is the ability to use all the heterogeneous resources in a given system. OpenCL makes it relatively straightforward to use all the devices in a node, including CPUs, GPUs and coprocessors such as Xeon Phi. The new version of BUDE presented in this paper requires very little performance from the host CPU, which only has to start the OpenCL kernels on the target device before being left to manage a very small amount of data transfer. We should therefore be able to harness the performance of the host as an OpenCL device itself, with negligible performance impact on the GPU devices in the system.

Finally, we believe this version of BUDE should exhibit strong scalability across many OpenCL devices, due to the porting of all of the remaining computation from the host CPU to the target devices. We plan on testing this multi-device scalability and reporting the results in a subsequent paper.

## 4.3 Conclusion

In this work, we have presented a highly optimized version of BUDE, a molecular docking application used for *in silico* virtual drug screening. Using OpenCL, we have achieved high levels of performance on an Nvidia GTX 680 GPU, which we believe can significantly improve the turn-around time of a virtual drug-design pipeline, whilst retaining state-of-the-art levels of accuracy. The sustained performance we have achieved of 46% of peak, or 1.43 TFLOP/s on a single GTX 680, is amongst the highest reported in the literature for a real application running on a GPU. We have also demonstrated strong performance portability across an architecturally diverse range of devices, from CPUs to GPUs, with an average efficiency across all eight devices in the study of 40%. We believe this is one of the first demonstrations of a real application that sustains a very high fraction of peak performance while being highly performance portable across a diverse range of many-core architectures. This result shows that the use of OpenCL combined with simple parameterization techniques can enable performance portable applications.
